# Coumarins as Powerful Photosensitizers for the Cationic Polymerization of Epoxy-Silicones under Near-UV and Visible Light and Applications for 3D Printing Technology

**DOI:** 10.3390/molecules25092063

**Published:** 2020-04-28

**Authors:** Mira Abdallah, Akram Hijazi, Frédéric Dumur, Jacques Lalevée

**Affiliations:** 1 Université de Haute-Alsace, CNRS, IS2M UMR 7361, F-68100 Mulhouse, France; 2 Université de Strasbourg, 67000 Strasbourg, France; 3 EDST, Université Libanaise, Campus Hariri, Hadath, Beyrouth, Liban; 4 Aix Marseille Univ, CNRS, ICR UMR 7273, F-13397 Marseille, France

**Keywords:** cationic polymerization, coumarin, epoxy-silicone, light-emitting diode, LED, photoinitiator, photopolymerization

## Abstract

In this study, eight coumarins (coumarins **1**–**8**) are proposed as near-UV and blue light sensitive photoinitiators/photosensitizers for the cationic polymerization (CP) of epoxysilicones when combined with 4-isopropyl-4’-methyldiphenyliodonium tetrakis(pentafluorophenyl)borate (IOD). Among these coumarins, four of them (coumarins **1**, **2**, **6** and **8**) have never been reported in the literature, i.e., these structures have been specifically designed to act as photoinitiators for silicones upon near UV and visible irradiation. Good final reactive epoxy function conversions (FCs) and also high rates of polymerization (Rp) were achieved in the presence of the newly proposed coumarin-based systems. The polymers generated from the photopolymerization of epoxysilicones can be considered as attractive candidates for several applications such as: elastomers, coatings, adhesives, and so on. The goal of this study focuses also on the comparison of the new proposed coumarins with well-established photosensitizers i.e., 1-chloro-4-propoxythioxanthone (CPTX), 9,10-dibutoxyanthracene (DBA) or some commercial coumarins (Com. Coum). As example of their high performance, the new proposed coumarins were also used for laser write experiments upon irradiation with a laser diode at 405 nm in order to develop new cationic 3D printing systems.

## 1. Introduction

Photopolymerization processes can occur via a radical process or an ionic process. Actually, photopolymers generated by the radical processes are usually characterized by high curing rates and often high rigidity. This type of polymerization also suffers from the oxygen inhibition issue but also from a rather high shrinkage during the polymerization process [1,2]. Conversely, cationic photopolymerization processes are insensitive to oxygen and low shrinkage is observed during the cationic process. However, lower curing rates are generally observed for this second type of polymerization process [3,4,5,6].

Light-induced cationic polymerization processes are more and more interesting for industry [7,8] due to the appealing properties that characterize the generated polymers ranging from their mechanical and chemical resistance properties to their great adhesion on support, their low shrinkage as well as their insensitivity toward oxygen under air [9,10]. Interestingly, for this type of polymerization, the reaction can continue even when the irradiation is turned off, phenomenon which is known as ‘‘dark polymerization’’. It results from the fact that the propagated cations do not react with oxygen. The cationic polymerization of several monomers such as vinyl ethers, epoxides, and so on, has been largely reported in the literature [11,12,13,14,15,16,17].

Crivello and coworkers have reported that epoxysilicone resins can be characterized by higher photopolymerization rates than the usual epoxy resins [18,19]. Curing of epoxysilicones also leads to polymers with interesting mechanical properties so that these resins are more and more studied [20,21].

**Figure 1:**
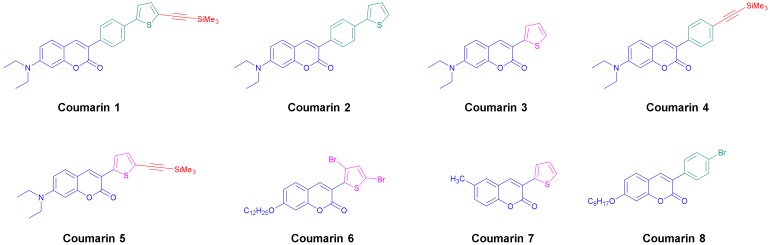
Chemical structures of the synthesized coumarins **1**–**8** used in this research.

In this work, the light-induced cationic polymerization of epoxy-silicone resins ((epoxycyclohexylethyl)methylsiloxane-dimethylsiloxane copolymer (Silcolease UV POLY 200: EPOX-Si200) has been studied in the presence of several blue light sensitive coumarins (Figure 1) combined with 4-isopropyl-4’-methyldiphenyliodonium tetrakis(pentafluorophenyl)borate (IOD also denoted as SpeedCure 939) as a photoinitiator, upon irradiation with a light-emitting diode (LED) at 405 nm. Excellent reactivities were shown in the presence of the new coumarin/IOD (0.05% or 0.1%/1% *w*/*w*) photoinitiating systems, highlighting the very high efficiencies of such compounds upon low power irradiation sources which is a challenging topic nowadays. Moreover, all the obtained photopolymers are tack-free directly after irradiation and a good adhesion on the support was also showed.

To evidence the pertinence of the approach, comparisons of the new proposed coumarin-based PISs with well-known photosensitizers (i.e., a thioxanthone derivative—CPTX and an anthracene derivative—DBA) but also with other commercial coumarins have been established in order to highlight the high initiating ability of coumarins **1**–**8**.

Parallel to this, 3D printing technologies are now widely used in biomedical engineering [22] radiology [23], 3D food printing [24], and many other fields. Various approaches can be followed to manufacture the 3D patterns, the most advantageous is the one that takes place through a photopolymerization process which has economic, environmental and also production interests. Therefore, new photocurable cationic formulations based on coumarins **1**–**8** have been developed in this work in order to generate thick 3D polymer samples via 3D printing experiments which were carried out under air and by means of a laser diode at 405 nm.

## 2. Results and Discussion

### 2.1. Synthesis of Coumarins

**Scheme 1:**
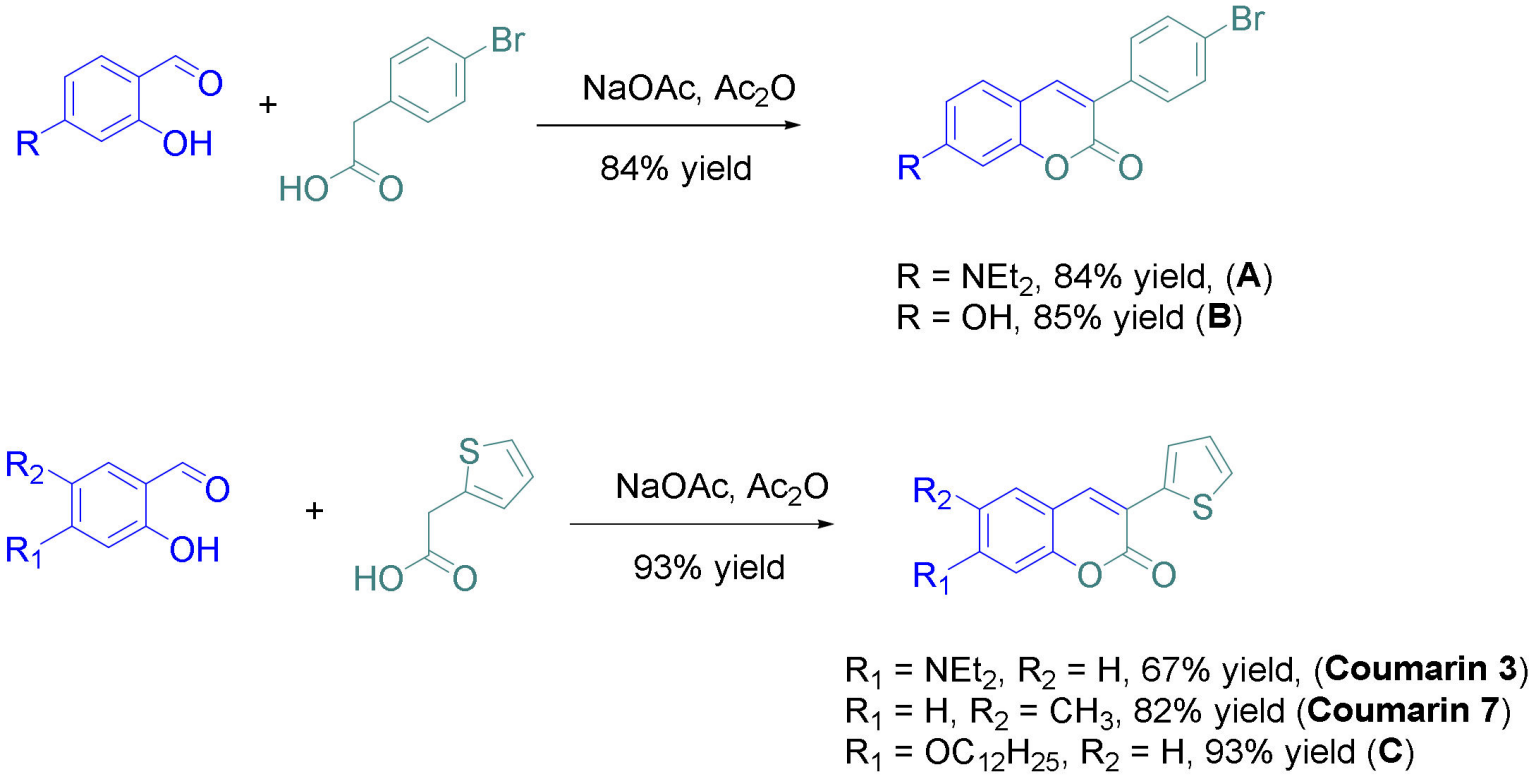
Synthetic routes to coumarins **3** and **7** and compounds **A**–**C**.

**Scheme 2:**
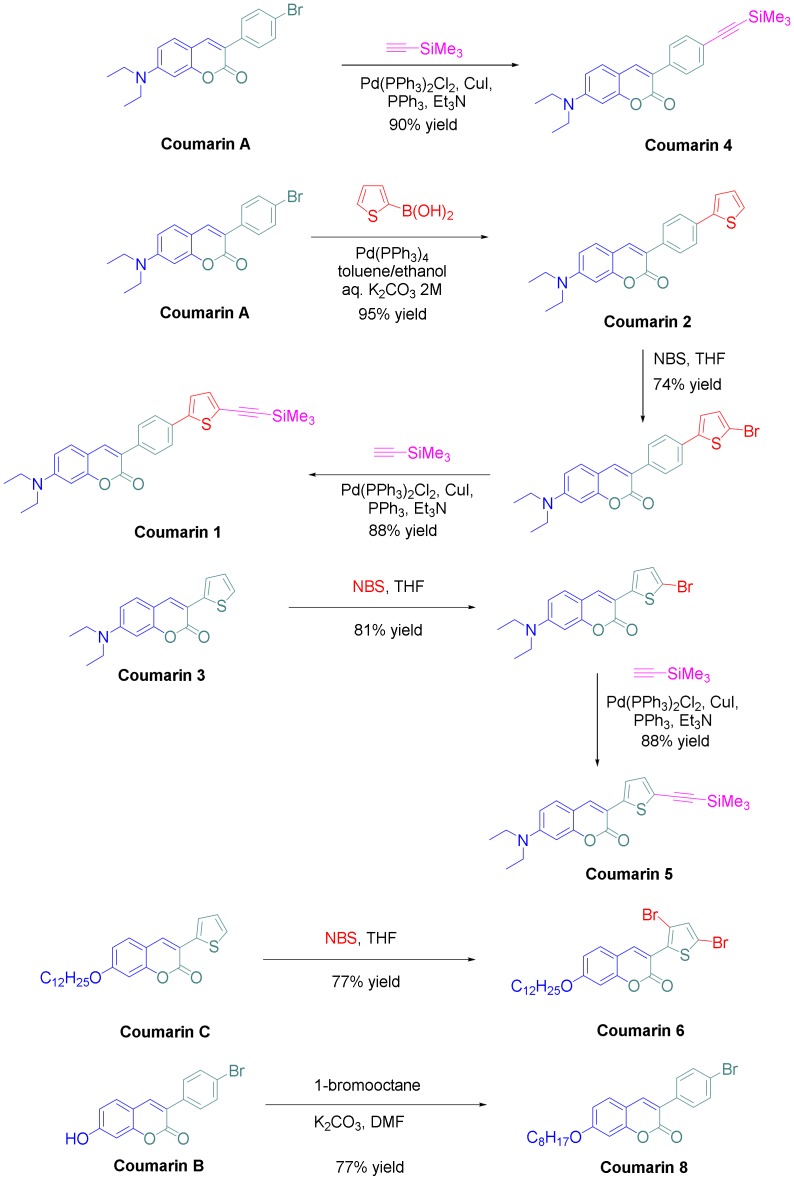
Synthetic routes to coumarins **1**, **2**, **4**–**6** and **8**.

Coumarins are chemical structures investigated since 1820 [25] and these derivatives are used in a wide range of applications ranging from medicine, biomedical research, and many industrial branches [26]. For this reason, many efforts have been dedicated to develop new practical methods for the synthesis of these compounds [27]. For easiness, only one procedure was used for the synthesis of the eight derivatives, consisting in the condensation of the appropriate salicylaldehyde derivative with the suitable phenylacetic acid derivative in acetic anhydride (see Scheme 1). All coumarins **A**–**C** and coumarins **3** and **7** could be obtained in yields ranging from 67 to 85%. Based on these structures, coumarins **1**, **2**, **4**–**6** and **8** could be prepared, following the synthetic routes reported in the Scheme 2 (see also the Supporting Information).

Thus, starting from coumarin **A**, coumarins **2** and **4** could be respectively obtained by a Suzuki cross-coupling reaction with 2-thiopheneboronic acid and a Sonogashira reaction with trimethylsilyl acetylene, providing the two molecules in yields of 95 and 90%, respectively. Coumarin **1** could be obtained in two steps starting from coumarin **2**, by bromination of the latter with *N*-bromo-succinimide (NBS) followed by a Sonogashira reaction with trimethylsilylacetylene. Coumarin **1** was obtained in pure form in 65% yield for the two steps. Using the same strategy than that used for coumarin **1**, coumarin **5** could be prepared in a higher reaction yield, reaching 71% for the two steps. Finally, coumarins **6** and **7** were synthesized in one step, by bromination of coumarin **C** with two equivalents of *N*-bromosuccinimide and by alkylation of coumarin **B** with 1-bromooctane under basic conditions. In turn, coumarins **6** and **7** were isolated in pure form in 77 and 77% yields, respectively. Among the eight coumarins reported in this work, four of them have never been reported in the literature and were specifically designed to act as photoinitiators.

### 2.2. Light Absorption Properties of the Examined Coumarins

**Figure 2:**
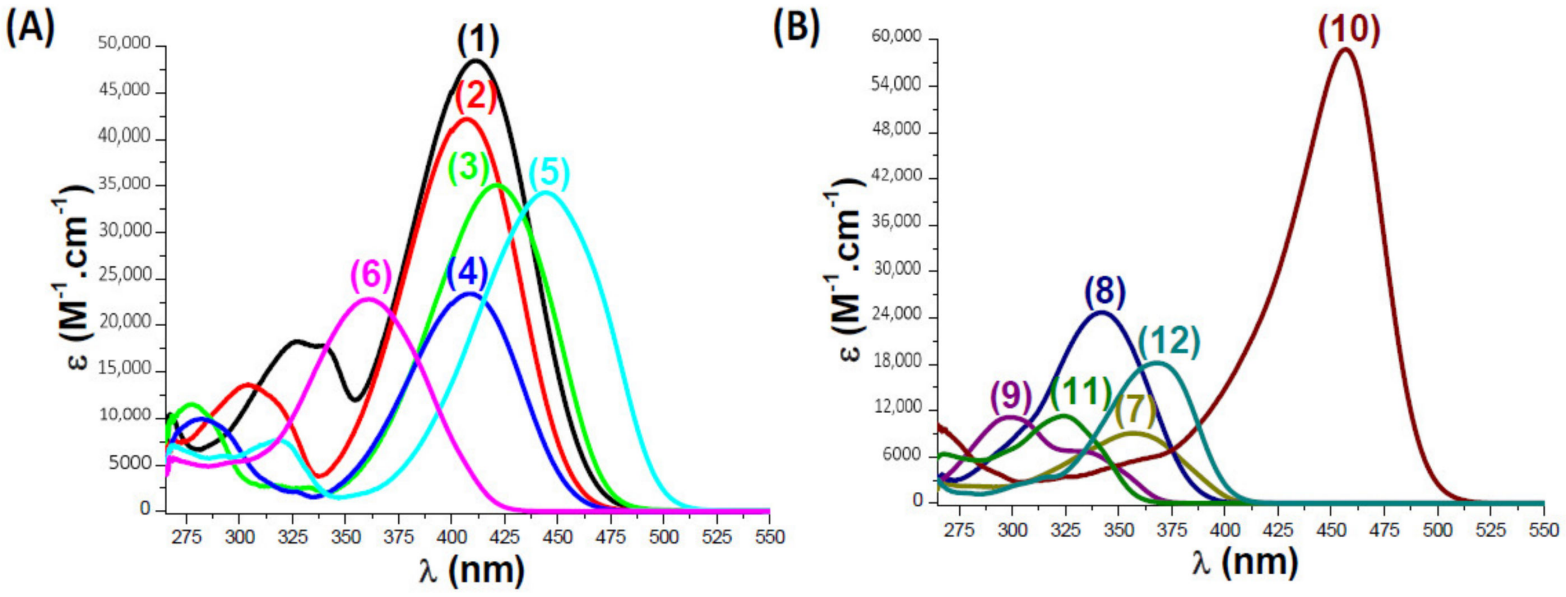
UV-Visible Absorption Spectra of Coumarins Studied in Acetonitrile: (**A**): (1) Coumarin **1**; (2) Coumarin **2**; (3) Coumarin **3**; (4) Coumarin **4**; (5) Coumarin **5**; (6) Coumarin **6**. (**B**): (7) Coumarin **7**; (8) Coumarin **8**; (9) Com. Coum **1**; (10) Com. Coum **2**; (11) Com. Coum **3**; (12) Com. Coum **4**, respectively.

The light absorption properties of the coumarins were studied in acetonitrile. As shown in Figure 2, the absorption spectra of the different coumarins are located in the near-UV and visible range allowing their use as near-UV and blue light sensitive photoinitiators. High molar extinction coefficients were determined for the synthesized coumarins within the 270–550 nm spectral range ensuring a good overlap with the emission spectra of the near-UV or visible LEDs. The molar extinction coefficients at λ**_max_** and also at the typical emission wavelength of the visible light source used in this study (LED@405 nm) are recapitulated in the Table 1.

### 2.3. Cationic Photopolymerization (CP) of Epoxy-Silicones

**Scheme 3:**
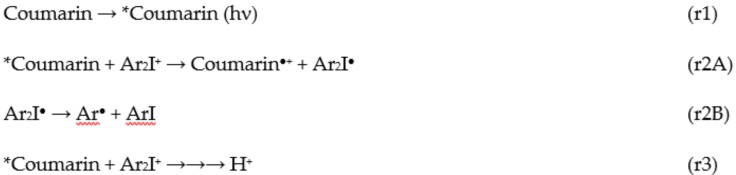
Proposed Photochemical Mechanisms for Cationic Photopolymerization Processes Initiated by Coumarins [13].

**Figure 3:**
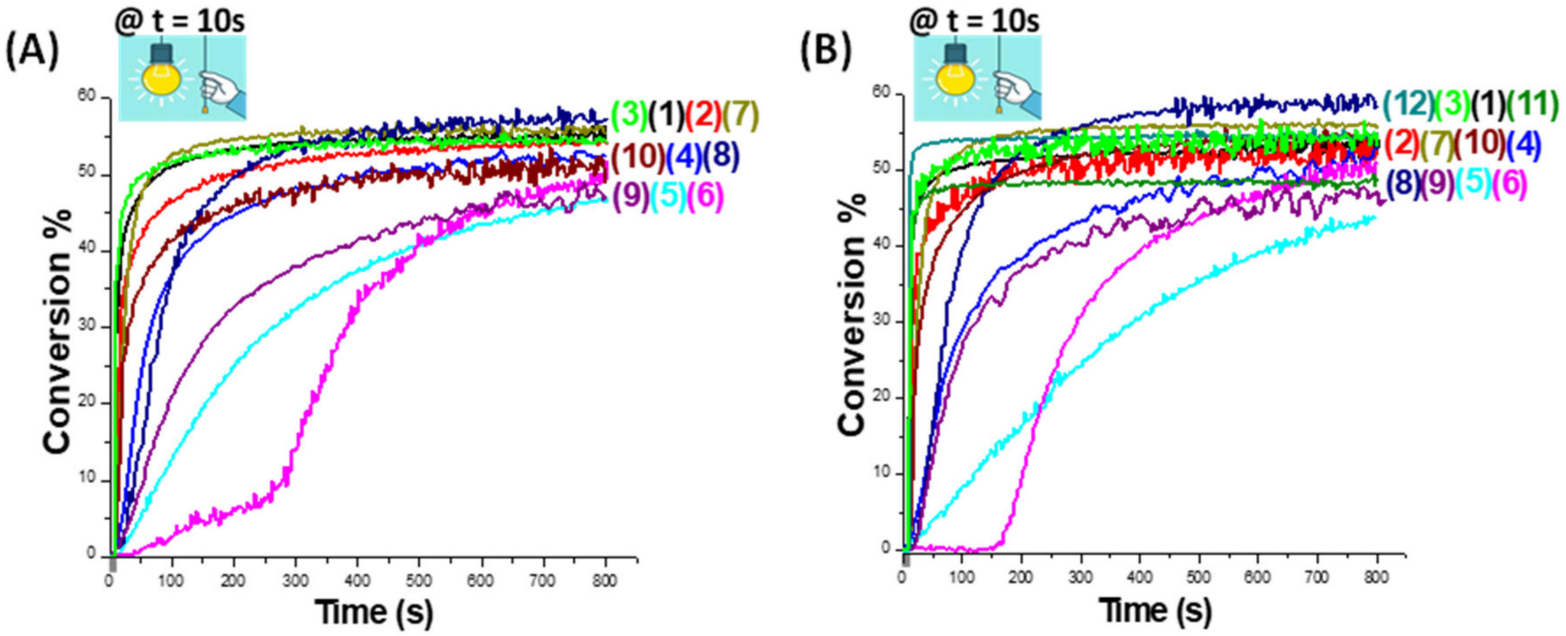
Polymerization profiles (epoxy function conversion vs. irradiation time) for thin epoxy-silicone films (thickness = 25 μm) under air, upon irradiation with the LED@405 nm, using different two-component photoinitiating systems: (**A**) **PI/IOD (0.05%/1% *w*/*w*):** (1–8) Coumarin **1-**Coumarin **8**/IOD; (9) Com. Coum **2**/IOD and (10) Com. Coum **4**/IOD, respectively. (**B**) **PI/IOD (0.1%/1% *w*/*w*):** (1–8) coumarin **1-**Coumarin **8**/IOD; (9) Com. Coum **2**/IOD; (10) Com. Coum **4**/IOD; (11) CPTX/IOD and (12) DBA/IOD, respectively. The irradiation starts at *t* = 10 s.

Actually, the investigated coumarins can efficiently absorb a blue light as aforementioned, then the corresponding generated excited states of such coumarins are expected to interact with IOD as a photoinitiator in order to generate reactive species (Coumarin^●+^; see reactions r1 and r2 in Scheme 3), which are able to initiate the CP of thin (25 μm) epoxy-silicone films (using EPOX-Si200 as the benchmarked monomer) upon exposure to the LED at 405 nm as shown in the Figure 3. The proposed photochemical mechanism for the coumarin/IOD interaction which occurs through the classical reduction of iodonium salt [28] is given in Scheme 3, and a full description of the mechanism has been already described by us in detail in [13,15]. The two-component coumarin/IOD (0.05%/1% *w*/*w*) photoinitiating systems are very efficient to initiate the CP under air where very high final functional conversions (FCs) and also high rates of polymerization (Rp) were achieved i.e., FC = 55% for coumarin **3**/IOD (0.05%/1% *w*/*w*) (curve 3 in Figure 3A; see also in Table 2 for the other coumarins). In fact, we mention that all the polymers obtained in this study are tack-free and well attached to the substrate. In addition, the generated photopolymers exhibit good bleaching properties after photocuring where the color of the generated polymers becomes lighter after irradiation (the color changes from yellow to transparent).

In these irradiation conditions, IOD (SC939) alone was tested and no polymerization occurs, highlighting the role of coumarins on the photoinitiating ability of these systems.

Interestingly, a better performance is observed in the presence of coumarins **1**, **2**, **3** and **7** when comparing to the commercial Com. Coum **4** (Figure 3A: curves 1–3, 7 vs. curve 10 for Com. Coum **4**/IOD (0.05%/1% *w*/*w*) system). The same holds true when higher concentrations of coumarins (0.1%) are used where an enhancement is noted in the performance of the system compared to the commercial coumarins Com. Coum **2** and Com. Coum **4** (Figure 3B). In addition, two other commercial coumarins Com. Coum **1** and Com. Coum **3** combined with IOD were tested and no efficiency was shown for the CP of epoxysilicones under the same irradiation conditions.

Furthermore, compared to well-established photosensitizers (e.g., CPTX or DBA), the new proposed systems are quite efficient as illustrated in Figure 3B (curves 11, 12 for CPTX and DBA vs. curves 1–3, 7 for the investigated synthesized coumarin **1**, coumarin **2**, coumarin **3** and coumarin **7**, respectively; see also in Table 3). The maximum reactive epoxy function conversion (FC) of epoxy-silicones reached was about 48% with (CPTX/IOD (0.1%/1% *w*/*w*)) and 55% with (DBA/IOD (0.1%/1% *w*/*w*)), a similar or even higher conversion was obtained with coumarins **1**, **2** and **3** (~55%) and 57% with coumarin **7**/IOD (0.1%/1% *w*/*w*).

Therefore, this comparison shows that the investigated coumarins can be interesting alternatives to commercial coumarins but also for benchmark photosensitizers such as CPTX or DBA. While comparing the two amounts of coumarins used (0.05% and 0.1%) in the photoinitiating systems, the two concentrations are quite efficient since both result in very high polymerization rates but also in high final reactive epoxy function conversions (FCs). Besides, a slight decrease in the polymerization rates at high concentrations is sometimes observed as shown in Figure 3B (with 0.1%) vs. Figure 3A (with 0.05%). This can be attributed to the lower solubility of coumarins at higher concentrations.

The efficiency trend for (coumarin/IOD) couples for CP (i.e., for the Rp) respects the following order: coumarin **3** > coumarin **1** > coumarin **2** > coumarin **7** > coumarin **4** > coumarin **8** > coumarin **5** > coumarin **6** which is directly linked to the light absorption properties of the examined coumarins (**ε_@405 nm_**), their photochemical reactivity (**Δ**G**_et(Coumarin/IOD)_**; see in Table 3), and also to the reactivity of the generated radical cations (Coumarin^●+^) which can have a huge effect on the photoinitiating ability. Furthermore, the reactivity of the system may also be affected by the formation of Brønsted acid which can be considered as the initiating species for the CP, as reported in [29]. Finally, the free energy changes (ΔG_et_) for the electron transfer reaction which occurs between coumarins as electron donors and IOD as electron acceptor (via singlet or triplet excited states) were calculated according to the equation 1 using the oxidation potentials E_ox_ (determined by cyclic voltammetry) and the excited state energies (E_S1_: the crossing point between the absorption and the fluorescence spectra or E_T1_: calculated triplet state energy level at DFT level) of the coumarins (Table 3).

### 2.4. 3D Patterns in the Presence of Coumarin/IOD Couples

The advantage of 3D printing experiments which occur through a cationic process compared to those which take place for a radical process is their ability to reduce the shrinkage usually observed in the latter case. At present, there are very few efficient photoinitiators for 3D applications via a cationic process of epoxysilicones. Therefore, the outstanding reactivity observed in cationic photopolymerization processes in the presence of coumarin/IOD PISs authorize their use for 3D printing applications via a cationic process.

**Figure 4:**
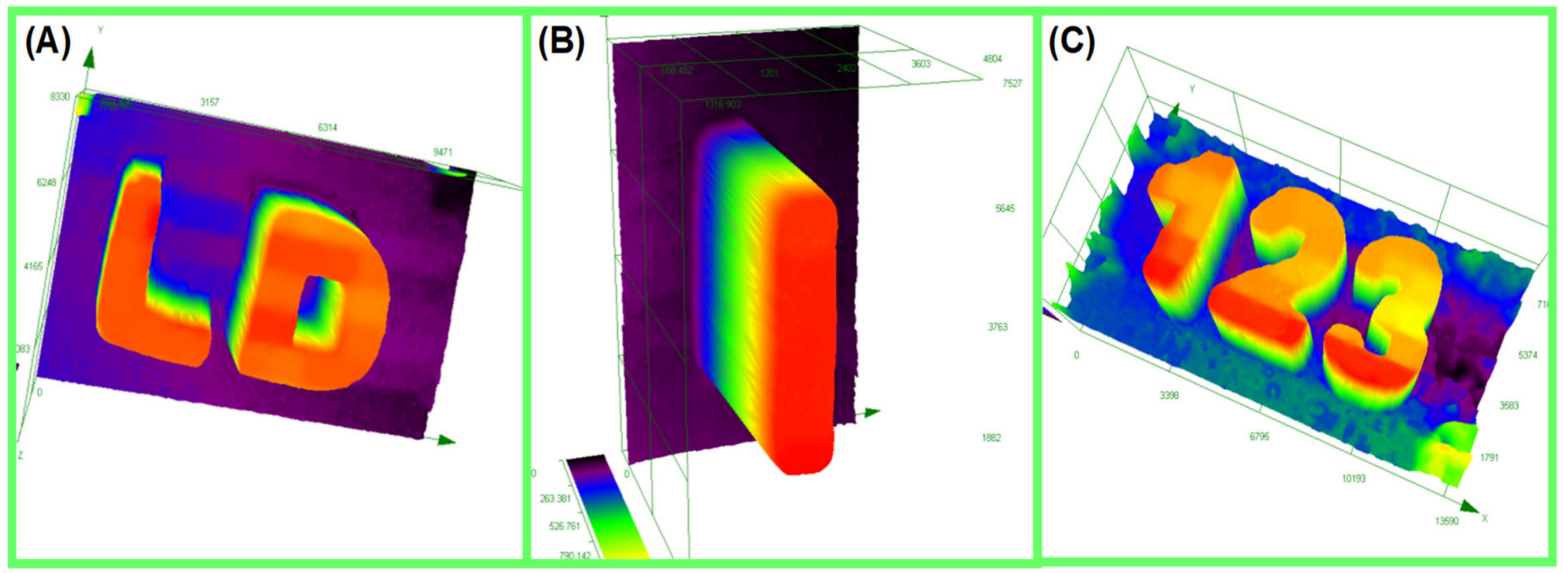
3D Printing Experiments concerning the CP of Epoxysilicones upon irradiation with a Laser Diode (@405 nm): Characterization of the thick 3D generated patterns by numerical optical microscopy: (**A**) Coumarin **2**/IOD (0.04%/0.8% *w*/*w*) (thickness = 1800 μm); (**B**) Coumarin **3**/IOD (0.018%/0.36% *w*/*w*) (thickness = 1320 μm) and **(C)** Coumarin **7**/IOD (0.05%/1% w/w) (thickness = 2200 μm); respectively.

EPOX-Si200 was used as the organic resin for this study. Thick 3D patterns with various thicknesses are generated by laser write experiments which were carried out under air with a laser diode at 405 nm. We mention that a very short time of irradiation (<1 min) was required to produce the 3D patterns. In addition, a very high spatial resolution was observed where this latter is only limited by the size of the laser diode beam: spot of 50 μm. Note that polymerization only occurs in the irradiated area, highlighting the high spatial control of the process. The generated thick 3D patterns are characterized by numerical optical microscopy (see Figure 4).

## 3. Materials and Methods

### 3.1. Synthesis of the Coumarins Investigated in This Research

The synthesis of coumarins **1**–**8** is discussed above in t Section 2.1 (see also the Supporting Information).

### 3.2. Commercial Chemical Compounds Used in This Study

**Scheme 4:**
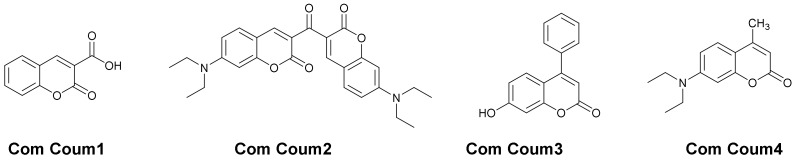
Chemical Structures of the Commercial Coumarins used in this Work.

**Scheme 5:**
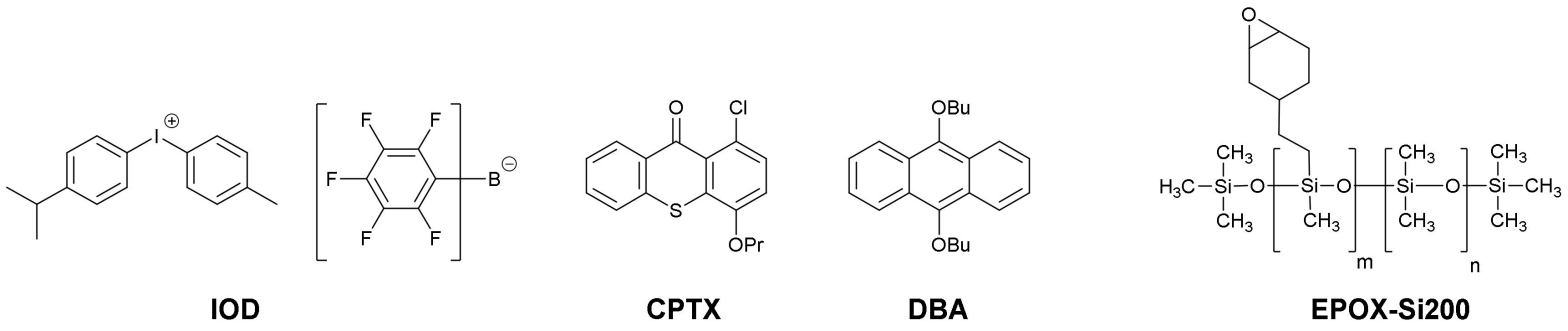
Chemical Structures of the Commercial Photoinitiators (IOD or SC939, CPTX and DBA) and Monomer (EPOX-Si200) used in this Study.

All commercial chemical compounds were selected with the highest purity available and used as received, and their chemical structures are presented in Scheme 4 and Scheme 5. The commercially available coumarins are the followings: 7-Hydroxy-4-phenylcoumarin (Com. Coum **3**) and 7-diethylamino-4-methylcoumarin (Com. Coum **4**) were obtained from Sigma Aldrich (Karlsruhe, Germany). Coumarin-3-carboxylic acid (Com. Coum **1**) and 3,3′-carbonyl-*bis*(7-diethylaminocoumarin) (Com. Coum **2**) were obtained from Tokyo Chemical Industry (TCI-europe, Bruxelle, Belgium). 4-Isopropyl-4’-methyldiphenyliodonium tetrakis(pentafluorophenyl)-borate (Iod or SpeedCure 939), 1-chloro-4-propoxythioxanthone (CPTX) and 9,10-dibutoxyanthracene (DBA) were obtained from Lambson Ltd. (Wetherby, UK) (Epoxycyclohexylethyl)methylsiloxane-dimethylsiloxane copolymer (Silcolease UV POLY 200: EPOX-Si200) was used as the benchmark epoxysilicone resin (obtained from Elkem-France, Lyon, France).

### 3.3. Light Irradiation Sources

The following Light Emitting Diode (LED): LED @405nm (I_0_ = 110 mW. cm^−2^) has been used as the blue light irradiation source for the photopolymerization processes. Otherwise, a laser diode (@405 nm) has been used for 3D printing experiments.

### 3.4. Cationic Photopolymerization (CP) Followed by Real-Time Fourier Transform InfraRed Spectroscopy (RT-FTIR)

In this work, different two-component photoinitiating systems (PISs) based on coumarin or commercial coumarin or CPTX or DBA/IOD (0.05% or 0.1%/1% *w*/*w*) couples have been tested for the cationic polymerization of epoxy-silicones. The weight percent of the different chemicals has been calculated from the monomer content (*w*/*w*). For the cationic polymerization, the different photosensitive formulations (thickness ~25 μm) are put on a BaF2 pellet under air, and the evolution of the epoxy group content of epoxy-silicones was continuously followed at about 3000 cm^−1^ by real time FTIR spectroscopy (FTIR 4100, JASCO, Tokyo, Japan). The procedure used to monitor the photopolymerization profiles has been already described in detail in [31,32,33].

### 3.5. Redox Potentials

The redox potentials (E_ox_ and E_red_) of the different investigated coumarins have been determined by cyclic voltammetry experiments. According to Equation (1) [34], the free energy changes (ΔG_et_) for the electron transfer reaction for the different examined coumarins were calculated. E_ox_, E_red_ and E* correspond to the oxidation potentials of the electron donor, the reduction potential of the electron acceptor and the excited state energy (E_S1_ or E_T1_) of the coumarin. C is the coulombic term for the initially formed ion pair which is usually neglected in polar solvents:

ΔG_et_ = E_ox_ − E_red_ − E* + C (1)

### 3.6. UV-Visible Absorption Experiments

The light absorption properties of the different coumarins used in this work were studied using a JASCO V730 UV–visible spectrometer.

### 3.7. Fluorescence Experiments

The fluorescence properties of the examined coumarins were studied using a JASCO FP-6200 spectrofluorimeter.

### 3.8. 3D Printing Experiments

In this study, different two-component photoinitiating systems (PISs) based on coumarin/IOD (x%/y% *w*/*w*) couples have been used for 3D printing experiments for cationic polymerization of epoxy-silicones. Indeed, the different photosensitive cationic resins (various thicknesses) are put in a mold. Then, a computer-controlled laser diode at 405 nm (spot size around 50 μm) is used as a light irradiation source for 3D polymerization process in order to generate 3D patterns under air. The characterization of the generated 3D patterns has been achieved using a numerical optical microscope (DSX-HRSU from OLYMPUS Corporation, Tokyo, Japan) as described by us in [35,36].

## 4. Conclusions

Here, new coumarins are incorporated into two-component (coumarin/IOD) photoinitiating systems in order to generate radical cations or acids which are able to efficiently initiate the cationic polymerization (CP) of epoxy-silicones upon blue light irradiation (LED@405 nm). These coumarin-based PISs can be interesting alternatives to well-known commercial coumarins or CPTX or DBA/IOD reference systems. The development of new cationic systems for 3D printing applications (upon laser diode @405 nm) was also accomplished in the presence of these coumarin-based PISs. Future development of new high-performance photoinitiating systems are under progress and will be presented in our upcoming papers.

## Figures and Tables

**Table 1:** Parameters Characterizing the Light Absorption Properties of the Synthesized and Commercial Coumarins used in this Study: Maximum Absorption Wavelengths λ_max_, Extinction Coefficients at λ_max_ and Extinction Coefficients @405 nm.

PI	λ_max_ (nm)	ε_max_ (M^−1^. cm^−1^)	ε_@405 nm_ (M^−1^. cm^−1^)
Coumarin **1**	411	48630	47950
Coumarin **2**	407	42360	42270
Coumarin **3**	421	35200	30600
Coumarin **4**	408	23530	23340
Coumarin **5**	445	34380	16880
Coumarin **6**	361	22970	6080
Coumarin **7**	357	9210	530
Coumarin **8**	342	24760	260
Com. Coum **1**	299	11150	40
Com. Coum **2**	457	58640	17450
Com. Coum **3**	324	11310	40
Com. Coum **4**	368	18200	1100

**Table 2:** Final Reactive Epoxy Function Conversion (FC) for Epoxy-silicones using Different Two-component PISs after 800 s of Irradiation with the LED @405 nm; thickness = 25 μm.

Coumarin 1	Coumarin 2	Coumarin 3	Coumarin 4	Coumarin 5	Coumarin 6	Coumarin 7	Coumarin 8	Com. Coum 2	Com. Coum 4	CPTX	DBA
57% ^a^	55% ^a^	55% ^a^	53% ^a^	47% ^a^	52% ^a^	57% ^a^	59% ^a^	47% ^a^	52% ^a^	48% ^b^	55% ^b^
55% ^b^	55% ^b^	55% ^b^	54% ^b^	44% ^b^	52% ^b^	57% ^b^	60% ^b^	47% ^b^	55% ^b^		

a: Coumarin or Com. Coum/IOD: (0.05%/1% *w*/*w*). b: Coumarin or Com. Coum or CPTX or DBA/IOD: (0.1%/1% *w*/*w*).

**Table 3:** Parameters Characterizing the Photochemical Mechanisms Associated with ^1,3^Coumarin/IOD in Acetonitrile.

PI	E_S1_ (eV)	E_T1_ (eV)^a^	E_ox_ (eV)	ΔG_et(S1)_^b^_(PI/Iod)_ (eV)	ΔG_et(T1)_^b^_(PI/Iod)_ (eV)
Coumarin **1**	2.7	1.94	1.03	−1.47	−0.71
Coumarin **2**	2.74	1.99	0.95	−1.59	−0.84
Coumarin **3**	2.68	1.81	0.81	−1.67	−0.8
Coumarin **4**	2.74	2.04	0.35	−2.19	−1.49
Coumarin **5**	2.55	1.67	0.45	−1.9	−1.02
Coumarin **6**	3.02	1.91	0.47	−2.35	−1.24
Coumarin **7**	2.69	1.81	1.12	−1.37	−0.49
Coumarin **8**	3.22	2.19	0.45	−2.57	−1.54

^a^ calculated triplet state energy level at DFT level (uB3LYP/6-31G* level of theory). ^b^ for Iod, a reduction potential of −0.2 eV was used for the ΔG_et_^1,3^(Coum/Iod) calculations [30].

## Supplementary Materials

The following are available online, Figure S1: Synthetic route to coumarin **1** and coumarin **2**, Figure S2: Synthetic route to coumarin **3** and coumarin **5**, Figure S3: Synthetic route to coumarin **6**, Figure S4: Synthetic route to coumarins **7** and **8**.
