# Supplementary material for: Coumarins as Powerful Photosensitizers for the Cationic Polymerization of Epoxy-Silicones under Near-UV and Visible Light and Applications for 3D Printing Technology
Source: Molecules. 2020 Apr 28;25(9):2063. doi: 10.3390/molecules25092063 (PMC7248746; doi:10.3390/molecules25092063)
Supplement: Supplementary file 1 [file molecules-25-02063-s001.pdf]

molecules

SUPPORTING INFORMATION

Coumarins as Powerful Photosensitizers for the Cationic Polymerization of Epoxy-Silicones under Near-UV and Visible Light and Applications for 3D Printing Technology

Mira Abdallah[1,2,3], Akram Hijazi[3], Frédéric Dumur[4,*] and Jacques Lalevée[1,2,*]

[1] Université de Haute-Alsace, CNRS, IS2M UMR 7361, F-68100 Mulhouse, France
[2] Université de Strasbourg, France
[3] EDST, Université Libanaise, Campus Hariri, Hadath, Beyrouth, Liban
[4] Aix Marseille Univ, CNRS, ICR UMR 7273, F-13397 Marseille, France
* Correspondence: frederic.dumur@univ.amu.fr, jacques.lalevee@uha.fr

Experimental part

All reagents and solvents were purchased from Aldrich or Alfa Aesar and used as received without further purification. Mass spectroscopy was performed by the Spectropole of Aix-Marseille University. ESI mass spectral analyses were recorded with a 3200 QTRAP (Applied Biosystems SCIEX) mass spectrometer. The HRMS mass spectral analysis was performed with a QStar Elite (Applied Biosystems SCIEX) mass spectrometer. Elemental analyses were recorded with a Thermo Finnigan EA 1112 elemental analysis apparatus driven by the Eager 300 software. ^{1}H and ^{13}C NMR spectra were determined at room temperature in 5 mm o.d. tubes on a Bruker Avance 400 spectrometer of the Spectropole: ^{1}H (400 MHz) and ^{13}C (100 MHz). The ^{1}H chemical shifts were referenced to the solvent peak DMSO (2.49 ppm) and the ^{13}C chemical shifts were referenced to the solvent peak DMSO (49.5 ppm). All these carbazole photoinitiators were prepared with analytical purity up to accepted standards for new organic compounds (>98%) which was checked by high field NMR analysis.

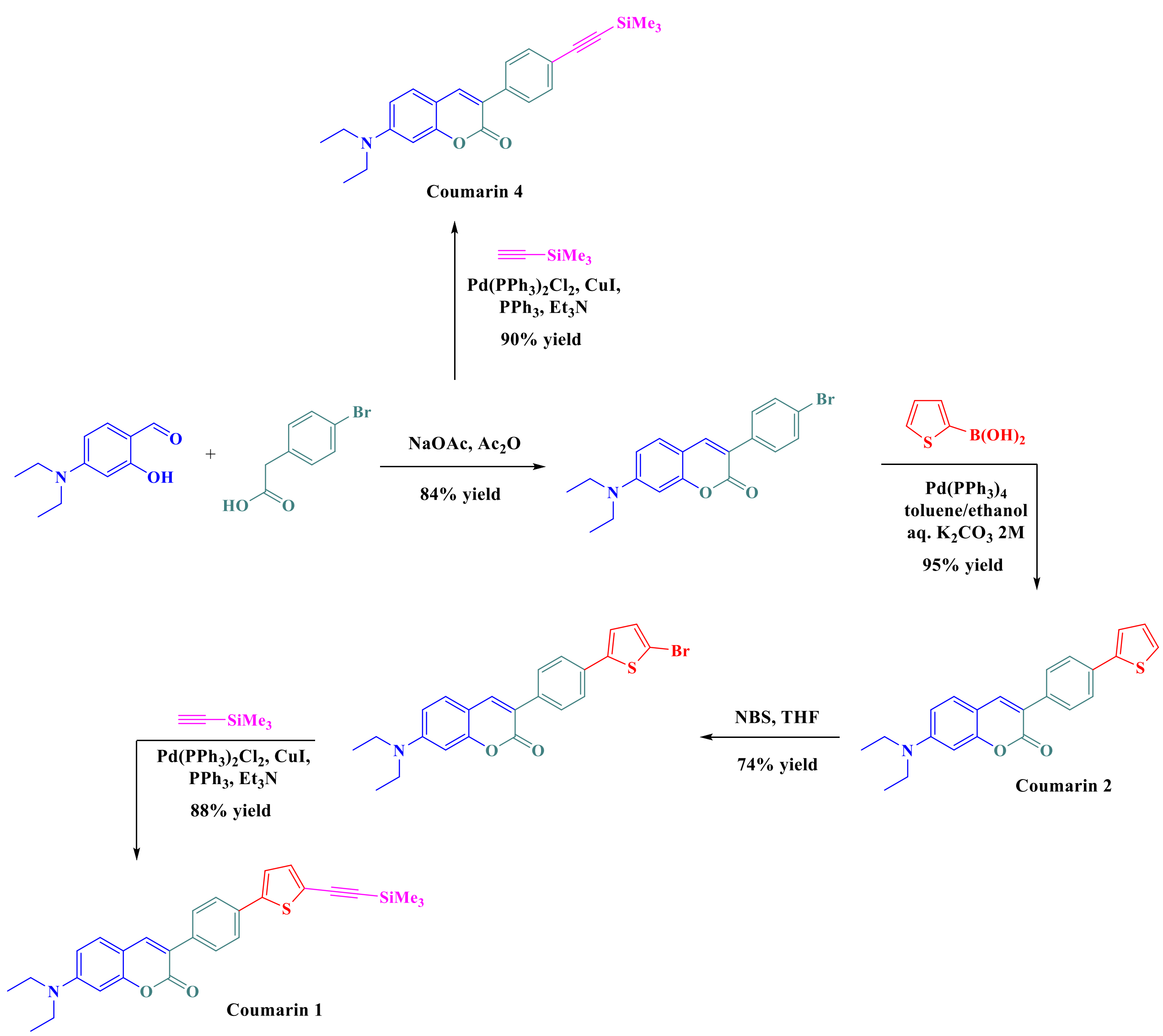

Figure S1. Synthetic route to **Coumarin 1** and **Coumarin 2**.

Synthesis of 3-(4-bromophenyl)-7-(diethylamino)-2H-chromen-2-one

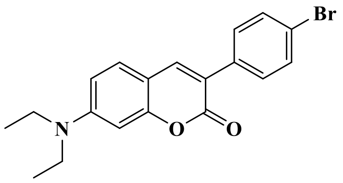

Chemical Formula: $C_{19}H_{18}BrNO_2$
Exact Mass: 371.0521
Molecular Weight: 372.2620

Anhydrous CH_3COONa (2.65 g, 32.07 mmol, M = 82.03 g/mol), 4-bromophenylacetic acid (4.96 g, 23.05 mmol, M = 215.05 g/mol), and 4-(diethylamino)salicylaldehyde (4.45 g, 23.05 mmol, M = 193.25 g/mol) was refluxed in Ac_2O (20 mL) for 2 h. The solution was cooled to room temperature during which time a precipitate formed. It was filtered off, washed with water and pentane. It was finally dried under vacuum (84% yield, 7.18 g). ^1H NMR ($CDCl_3$) δ : 1.23 (t, 6H, J = 1.1 Hz), 3.43 (q, 4H, J =

1.1 Hz), 6.52 (d, 1H, J = 2.1 Hz), 6.60 (dd, 1H, J = 8.8 Hz, J = 2.1 Hz), 7.31 (d, 1H, J = 8.8 Hz), 7.52 (d, 2H, J = 8.5 Hz), 7.59 (d, 2H, J = 8.5 Hz), 7.69 (s, 1H); ^{13}C NMR ($CDCl_3$) δ : 12.5, 44.9, 97.1, 108.9, 109.1, 119.5, 121.7, 129.0, 129.8, 131.4, 134.8, 140.6, 150.7, 156.3, 161.4; HRMS (ESI MS) m/z: theor: 372.0594 found: 372.0595 ($[M+H]^+$ detected).

*Synthesis of 7-(diethylamino)-3-(4-(thiophen-2-yl)phenyl)-2H-chromen-2-one (**Coumarin 2**)*

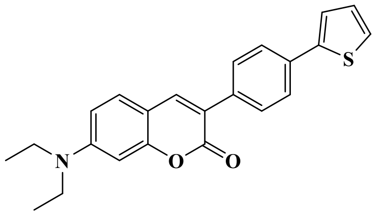

Chemical Formula: $C_{23}H_{21}NO_2S$
Exact Mass: 375.1293
Molecular Weight: 375.4860

Tetra*kis*(triphenylphosphine)palladium (0) (0.46 g, 0.744 mmol, M = 1155.56 g.mol^{-1}) was added to a mixture of 3-(4-bromophenyl)-7-(diethylamino)-2*H*-chromen-2-one (2.27 g, 6.11 mmol, M = 372.26 g.mol^{-1}), 2-thiopheneboronic acid (1.18 g, 9.16 mmol, M = 127,96 g.mol^{-1}), toluene (54 mL), ethanol 26 mL) and an aqueous potassium carbonate solution (2 M, 6.91 g in 25 mL water, 26 mL) under vigorous stirring. The mixture was stirred at 80 °C for 48 h under a nitrogen atmosphere. After cooling to room temperature, the reaction mixture was poured into water and extracted with ethyl acetate. The organic layer was washed with brine several times, and the solvent was then evaporated. Addition of DCM followed by pentane precipitated a white solid which was filtered off. The residue was purified by column chromatography (SiO_2, pentane/DCM: 1/1 and pure DCM) and isolated as a white solid (95% yield, 2.18 g). ^{1}H NMR ($CDCl_3$) δ : 1.25 (t, 6H, J = 7.1 Hz), 3.46 (q, 4H, J = 7.1 Hz), 6.57 (d, 1H, J = 2.1 Hz), 6.63 (dd, 1H, J = 8.8 Hz, J = 2.1 Hz), 7.12 (t, 1H, J = 5.0 Hz), 7.32 (d, 1H, J = 5.0 Hz), 7.34-7.37 (m, 2H), 7.68 (d, 2H, J = 8.4 Hz), 7.75-7.77 (m, 3H); ^{13}C NMR ($CDCl_3$) δ : 12.5, 44.9, 97.3, 109.1, 109.2, 120.3, 123.2, 124.9, 125.8, 128.0, 128.6, 129.0, 133.8, 134.9, 140.1, 144.1, 150.5, 156.2, 161.5 ; HRMS (ESI MS) m/z: theor: 376.1366 found: 376.1364 ($[M+H]^+$ detected).

*Synthesis of 7-(diethylamino)-3-(4-((trimethylsilyl)ethynyl)phenyl)-2H-chromen-2-one (**Coumarin 4**)*

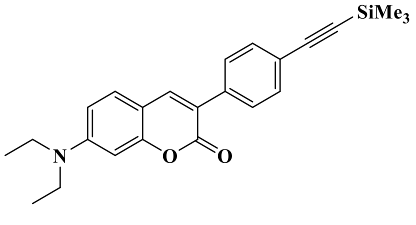

Chemical Formula: $C_{24}H_{27}NO_2Si$
Exact Mass: 389.1811
Molecular Weight: 389.5700

To 3-(4-bromophenyl)-7-(diethylamino)-2*H*-chromen-2-one (331 mg, 0.89 mmol, M = 372.26 g/mol) was added $Pd(PPh_3)_2Cl_2$ (63 mg, 0.089 mmol), CuI (17 mg, 0.089 mmol), PPh_3 (48 mg, 0.180 mmol)

and trimethylsilylacetylene (378 mL, 2.67 mmol). NEt_3 (5 mL) and THF (25 mL) were then added and the reaction mixture was refluxed for 12 h under inert atmosphere. After reaction, the solvent was removed under reduced pressure and the crude residue was purified by column chromatography (1:6 v/v ethyl acetate/ hexane) to give the product as yellow solid (90% yield, 312 mg). ^1H NMR ($CDCl_3$) δ : 0.19 (s, 9H), 1.15 (t, 6H, J = 7.0 Hz), 3.35 (q, 4H, J = 7.0 Hz), 6.45 (d, 1H, J = 1.9 Hz), 6.52 (dd, 1H, J = 8.8 Hz, J = 1.9 Hz), 7.23 (d, 1H, J = 8.8 Hz), 7.42 (d, 2H, J = 8.2 Hz), 7.60 (d, 2H, J = 8.2 Hz), 7.64 (s, 1H); ^{13}C NMR ($CDCl_3$) δ : 0.0, 12.5, 44.9, 94.9, 97.1, 105.1, 109.0, 109.1, 119.8, 122.3, 127.9, 128.5, 128.6, 129.1, 131.9, 133.8, 134.0, 135.9, 140.6, 150.7, 156.3, 161.3; HRMS (ESI MS) m/z: theor: 390.1884 found: 390.1888 ($[M+H]^+$ detected).

Synthesis of 3-(4-(5-bromothiophen-2-yl)phenyl)-7-(diethylamino)-2H-chromen-2-one

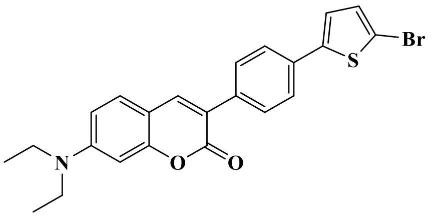

Chemical Formula: $C_{23}H_{20}BrNO_2S$
Exact Mass: 453.0398
Molecular Weight: 454.3820

N-Bromosuccinimide (2.6 g, 14.69 mmol, M = 177.98 g/mol) was added in small portions to a solution of 7-(diethylamino)-3-(4-(thiophen-2-yl)phenyl)-2*H*-chromen-2-one (5.0 g, 13.36 mmol, M = 375.49 g/mol) in THF (120 mL) at room temperature. The mixture was stirred for 4 hours, and water (20 mL) was added. The mixture was extracted with CH_2Cl_2 several times. The combined organic phases were washed with water (100 mL), dried over magnesium sulfate and the solvent removed under reduced pressure. The residue was suspended in a minimum of ether and addition of pentane precipitated a yellow solid that was filtered off, washed several times with pentane and dried under vacuum (74% yield, 4.49 g). ^1H NMR ($CDCl_3$) δ : 1.22 (t, 6H, J = 7.1 Hz), 3.42 (q, 4H, J = 7.1 Hz), 6.52 (d, 1H, J = 2.3 Hz), 6.60 (dd, 1H, J = 8.8 Hz, J = 2.3 Hz), 7.02 (d, 1H, J = 3.9 Hz), 7.07 (d, 1H, J = 3.9 Hz), 7.31 (d, 1H, J = 6.3 Hz), 7.52 (1H, J = 8.5 Hz), 7.63-7.65 (m, 1H), 7.69-7.74 (m, 3H); ^{13}C NMR ($CDCl_3$) δ : 12.5, 44.9, 97.1, 109.1, 111.5, 119.9, 123.3, 125.4, 125.8, 128.6, 129.0, 130.9, 132.8, 135.4, 140.2, 145.6, 150.7, 156.3, 161.5; HRMS (ESI MS) m/z: theor: 454.0471 found: 454.0476 ($[M+H]^+$ detected).

*Synthesis of 7-(Diethylamino)-3-(5-((trimethylsilyl)ethynyl)thiophen-2-yl)-2H-chromen-2-one (**Coumarin 1**)*

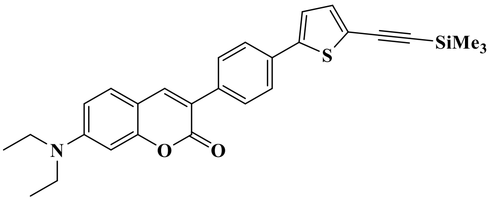

Chemical Formula: $C_{28}H_{29}NO_2SSi$
Exact Mass: 471.1688
Molecular Weight: 471.6900

To 3-(4-(5-bromothiophen-2-yl)phenyl)-7-(diethylamino)-2*H*-chromen-2-one (404 mg, 0.89 mmol, M = 454.38 g/mol) was added $Pd(PPh_3)_2Cl_2$ (63 mg, 0.089 mmol), CuI (17 mg, 0.089 mmol), PPh_3 (48 mg, 0.180 mmol) and trimethylsilylacetylene (378 mL, 2.67 mmol). NEt_3 (5 mL) and THF (25 mL) were then added and the reaction mixture was refluxed for 12 h under inert atmosphere. After reaction, the solvent was removed under reduced pressure and the crude residue was purified by column chromatography (1:6 v/v ethyl acetate/ hexane) to give the product as yellow solid (88% yield, 369 mg). ^{1}H NMR ($CDCl_3$) δ : 0.29 (s, 9H), 1.25 (t, 6H, J = 7.1 Hz), 3.45 (q, 4H, J = 7.1 Hz), 6.56 (d, 1H, J = 2.0 Hz), 6.63 (dd, 1H, J = 8.7 Hz, J = 2.0 Hz), 7.21 (d, 1H, J = 3.8 Hz), 7.22 (d, 1H, J = 3.8 Hz), 7.34 (d, 1H, J = 8.8 Hz), 7.62 (d, 1H, J = 8.4 Hz), 7.73-7.77 (m, 3H); ^{13}C NMR ($CDCl_3$) δ : -0.14, 12.5, 44.9, 97.2, 97.7, 99.7, 109.1, 109.2, 120.0, 122.4, 122.9, 125.7, 125.8, 125.9, 128.7, 129.0, 132.9, 133.7, 135.5, 140.2, 145.5, 150.6, 156.3, 161.4; HRMS (ESI MS) m/z: theor: 472.1761 found: 472.1761 ($[M+H]^+$ detected).

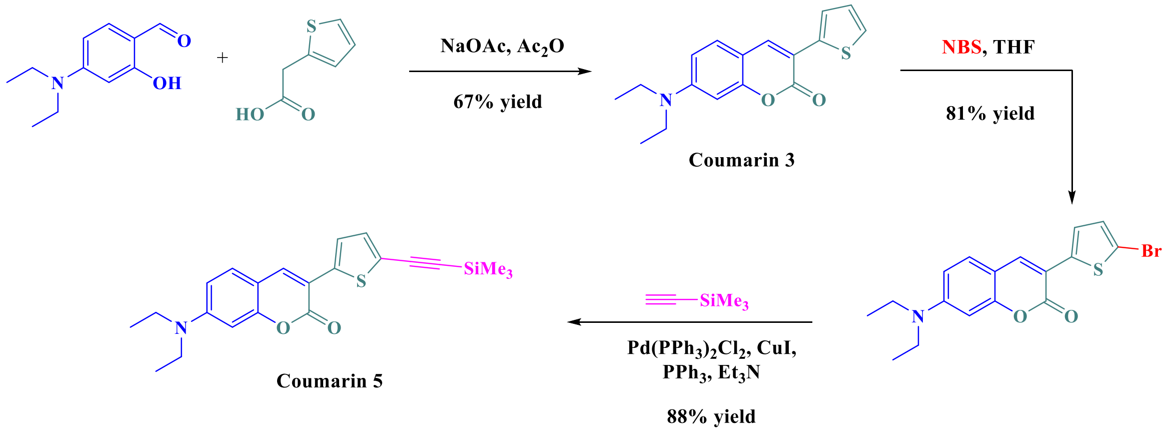

Figure S2. Synthetic route to **Coumarin 3** and **Coumarin 5**.

*Synthesis of 7-(diethylamino)-3-(thiophen-2-yl)-2H-chromen-2-one (**Coumarin 3**)*

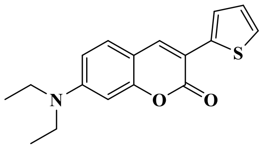

Chemical Formula: $C_{17}H_{17}NO_2S$
Exact Mass: 299.0980
Molecular Weight: 299.3880

Thiophene acetic acid (7.80 g, 55 mmol, M = 142.17 g/mol), 4-(diethylamino)salicylaldehyde (16.4 g, 85 mmol, M = 193.24 g/mol) were dissolved in acetic anhydride (200 mL). Triethylamine (14.6 mL, 105 mmol) was added and the mixture was stirred at reflux for three hours. The reaction was cooled down at room temperature, water was added, the organic material was extracted with AcOEt (3 × 50 mL) and the organic layers were dried over magnesium sulfate. The solvent was removed under reduced pressure and the residue was purified by flash column chromatography (SiO_2, DCM:pentane 1:1) to afford the coumarin as a yellow solid (67% yield, 11.03 g). ^{1}H NMR ($CDCl_3$) δ : 1.18 (t, 6H, J = 7.1 Hz), 3.39 (q, 4H, J = 7.1 Hz), 6.50 (d, 1H, J = 2.4 Hz), 6.57 (dd, 1H, J = 8.8 Hz, J = 2.4 Hz), 7.04 (t, 1H, J = 3.8 Hz), 7.26-7.29 (m, 2H), 7.62 (d, 1H, J = 3.7 Hz), 7.83 (s, 1H); ^{13}C NMR ($CDCl_3$) δ : 12.5, 44.9, 97.2, 108.8, 109.3, 114.9, 124.9, 125.5, 127.2, 128.8, 136.8, 137.5, 150.5, 155.6, 160.5; HRMS (ESI MS) m/z: theor: 300.1053 found: 300.1051 ($[M+H]^+$ detected).

Synthesis of 3-(5-bromothiophen-2-yl)-7-(diethylamino)-2H-chromen-2-one

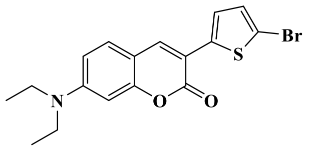

Chemical Formula: $C_{17}H_{16}BrNO_2S$
Exact Mass: 377.0085
Molecular Weight: 378.2840

NBS (2.6 g, 14.69 mmol, M = 177.98 g/mol) was added in small portions to a solution of 7-(diethylamino)-3-(thiophen-2-yl)-2H-chromen-2-one (4.0 g, 13.36 mmol, M = 299.39 g/mol) in THF (120 mL) at room temperature. The mixture was stirred for 4 hours, and water (20 mL) was added. The mixture was extracted with CH_2Cl_2 several times. The combined organic phases were washed with water (100 mL), dried over magnesium sulfate and the solvent removed under reduced pressure. The residue was suspended in a minimum of ether and addition of pentane precipitated a yellow solid that was filtered off, washed several times with pentane and dried under vacuum (81% yield, 4.09 g). ^{1}H NMR ($CDCl_3$) δ : 1.21 (t, 6H, J = 7.1 Hz), 3.43 (q, 4H, J = 7.1 Hz), 6.52 (d, 1H, J = 1.9 Hz), 6.62 (dd, 1H, J = 8.8 Hz, J = 1.9 Hz), 7.03 (d, 1H, J = 4.0 Hz), 7.30-7.33 (m, 2H), 7.82 (s, 1H); ^{13}C NMR ($CDCl_3$) δ : 12.5, 44.9, 97.2, 108.6, 109.5, 113.2, 114.1, 124.0, 129.7, 136.1, 138.7, 150.7, 155.5, 160.5; HRMS (ESI MS) m/z: theor: 378.0158 found: 378.0160 ($[M+H]^+$ detected).

*Synthesis of 7-(diethylamino)-3-(5-((trimethylsilyl)ethynyl)thiophen-2-yl)-2H-chromen-2-one (**Coumarin 5**)*

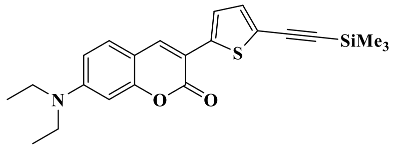

Chemical Formula: $C_{22}H_{25}NO_2SSi$
Exact Mass: 395.1375
Molecular Weight: 395.5920

To 3-(5-bromothiophen-2-yl)-7-(diethylamino)-2*H*-chromen-2-one (336 mg, 0.89 mmol, M = 378.28 g/mol) was added $Pd(PPh_3)_2Cl_2$ (63 mg, 0.089 mmol), CuI (17 mg, 0.089 mmol), PPh_3 (48 mg, 0.180 mmol) and trimethylsilylacetylene (378 mL, 2.67 mmol). NEt_3 (5 mL) and THF (25 mL) were then added and the reaction mixture was refluxed for 12 h under an inert N_2 environment. After the reaction, solvent was removed and the crude residue was purified by column chromatography (1:6 v/v ethyl acetate/ hexane) to give the product as yellow solid (88% yield, 310 mg). ^{1}H NMR ($CDCl_3$) δ : 0.25 (s, 9H), 1.22 (t, 6H, J = 7.1 Hz), 3.43 (q, 4H, J = 7.1 Hz), 6.51 (d, 1H, J = 2.3 Hz), 6.61 (dd, 1H, J = 8.8 Hz, J = 2.3 Hz), 7.18 (d, 1H, J = 3.9 Hz), 7.46 (d, 1H, J = 3.9 Hz), 7.85 (s, 1H) ; ^{13}C NMR ($CDCl_3$) δ : -0.1, 12.5, 44.9, 97.2, 98.0, 99.9, 108.7, 109.4, 114.1, 123.0, 124.0, 128.4, 128.5, 129.0, 129.7, 132.7, 134.1, 134.2, 136.9, 138.9, 150.8, 155.7, 160.4; HRMS (ESI MS) m/z: theor: 396.1448 found: 396.1444 ($[M+H]^+$ detected).

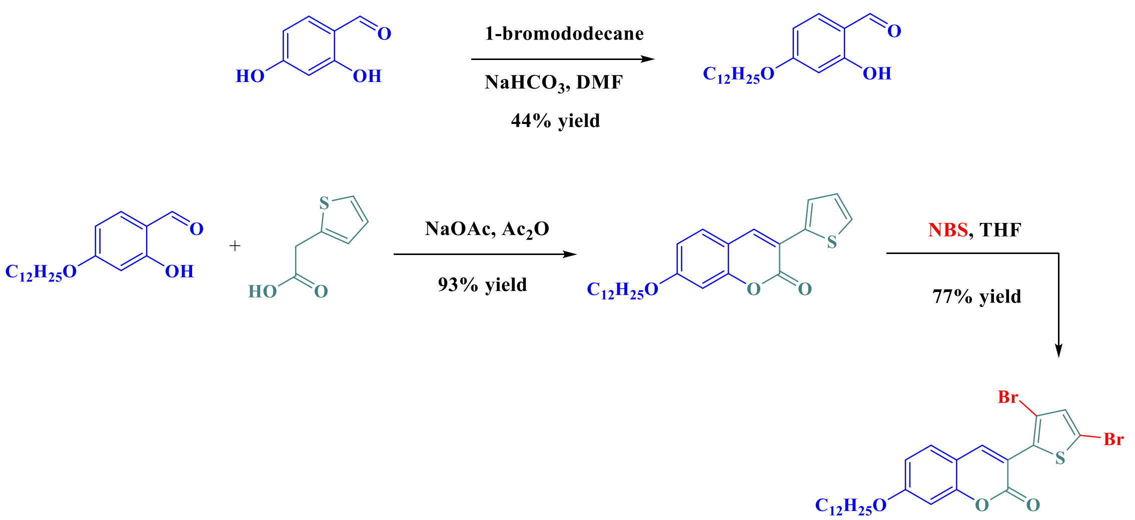

Coumarin 6

Figure S3. Synthetic route to **Coumarin 6**.

Synthesis of 4-dodecyloxy-2-hydroxybenzaldehyde

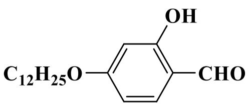

Chemical Formula: $C_{19}H_{30}O_3$
Exact Mass: 306.2195
Molecular Weight: 306.4460

2,4-Dihydroxybenzaldehyde (2.76 g, 20 mmol, M = 138.12 g/mol) and sodium hydrogenocarbonate (1.68 g, 20 mmol, M = 84.0 g/mol) were dissolved in DMF (60 mL). Then, 1-bromododecane (4.98 g, 4.80 mL, 20 mmol, M = 249.23 g/mol, d = 1.038) was added and heated at 100°C overnight. The solution was concentrated to dryness to give a mixture as a red-brown oil. The residue was purified by column chromatography (SiO_2) using DCM as the eluent (44% yield, 2.70 g). ^{1}H NMR ($CDCl_3$) δ : 0.88 (t, 3H, J = 6.5 Hz), 1.21-1.47 (m, 19H), 1.82 (qt, 2H, J = 7.9 Hz), 4.00 (t, 2H, J = 6.6 Hz), 6.41 (d, 1H, J = 2.3 Hz), 6.52 (dd, 1H, J = 8.7 Hz, J = 2.3 Hz), 7.41 (d, 1H, J = 8.7 Hz), 9.70 (s, 1H), 11.46 (s, 1H); ^{13}C NMR ($CDCl_3$) δ : 14.1, 22.7, 25.9, 28.92, 29.28, 29.32, 29.51, 29.55, 29.60, 29.62, 31.9, 68.6, 101.1, 108.8, 115.0, 135.2, 164.5, 166.5, 194.2; HRMS (ESI MS) m/z: theor: 307.2268 found: 307.2272 ($[M+H]^+$ detected).

Synthesis of 7-dodecyloxy-3-(thiophen-2-yl)-2H-chromen-2-one

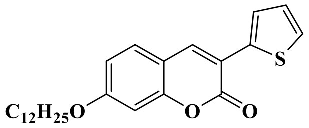

Chemical Formula: $C_{25}H_{32}O_3S$
Exact Mass: 412.2072
Molecular Weight: 412.5880

Anhydrous CH_3COONa (2.65 g, 32.07 mmol, M = 82.03 g/mol), 2-thiopheneacetic acid (3.28 g, 23.05 mmol, M = 142.17 g/mol), and 4-dodecyloxy-2-hydroxybenzaldehyde (7.06 g, 23.05 mmol, M = 306.45

g/mol) was refluxed in Ac_2O (20 mL) overnight. The solution was cooled to room temperature during which time a precipitate formed. It was filtered off, washed with water and pentane and dried under vacuum (93% yield, 8.84 g). ^{1}H NMR ($CDCl_3$) δ : 0.88 (t, 3H, J = 6.4 Hz), 1.27-1.55 (m, 20H), 1.82 (qt, 2H, J = 6.6 Hz), 4.02 (t, 2H, J = 6.5 Hz), 6.84-6.89 (m, 2H), 7.10 (t, 1H, J = 3.8 Hz), 7.38 (d, 1H, J = 5.1 Hz), 7.43 (d, 1H, J = 8.5 Hz), 7.73 (dd, 1H, J = 3.7 Hz, J = 0.9 Hz), 7.94 (s, 1H); ^{13}C NMR ($CDCl_3$) δ : 14.1, 22.7, 26.0, 29.0, 29.3, 29.5, 29.57, 29.62, 29.64, 31.9, 100.9, 112.8, 113.5, 118.4, 126.2, 126.7, 127.4, 128.6, 136.1, 136.6, 154.6, 159.8, 162.2; HRMS (ESI MS) m/z: theor: 413.2145 found: 413.2144 ($[M+H]^+$ detected).

*Synthesis of 3-(3,5-dibromothiophen-2-yl)-7-(dodecyloxy)-2H-chromen-2-one (**Coumarin 6**)*

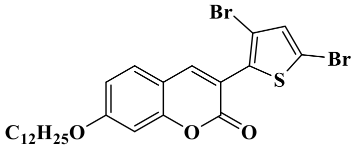

Chemical Formula: $C_{25}H_{30}Br_2O_3S$
Exact Mass: 568.0282
Molecular Weight: 570.3800

NBS (5.2 g, 29.38 mmol, M = 177.98 g/mol) was added in small portions to a solution of 7-(dodecyloxy)-3-(thiophen-2-yl)-2*H*-chromen-2-one (5.51 g, 13.36 mmol, M = 412.59 g/mol) in THF (120 mL) at room temperature. The mixture was stirred for 4 hours, and water (20 mL) was added. The mixture was extracted with CH_2Cl_2 several times. The combined organic phases were washed with water (100 mL), dried over magnesium sulfate and the solvent removed under reduced pressure. The residue was suspended in a minimum of ether and addition of pentane precipitated a yellow solid that was filtered off, washed several times with pentane and dried under vacuum (77% yield, 5.87 g). ^{1}H NMR ($CDCl_3$) δ : 0.88 (t, 3H, J = 6.6 Hz), 1.21-1.42 (m, 16H), 1.45 (qt, 2H, J = 7.6 Hz), 1.82 (qt, 2H, J = 6.4 Hz), 4.03 (t, 2H, J = 6.6 Hz), 6.84 (d, 1H, J = 2.2 Hz), 6.89 (dd, 1H, J = 8.7 Hz, J = 2.2 Hz), 7.04 (s, 1H), 7.46 (d, 1H, J = 8.7 Hz), 8.3 (s, 1H); ^{13}C NMR ($CDCl_3$) δ : 14.1, 22.7, 25.9, 28.9, 29.31, 29.33, 29.57, 29.62, 29.64, 31.9, 68.9, 101.1, 108.8, 112.2, 113.8, 114.9, 116.1, 129.4, 132.0, 132.8, 141.4, 155.2, 160.1, 163.1; HRMS (ESI MS) m/z: theor: 569.0365 found: 569.0367 ($[M+H]^+$ detected).

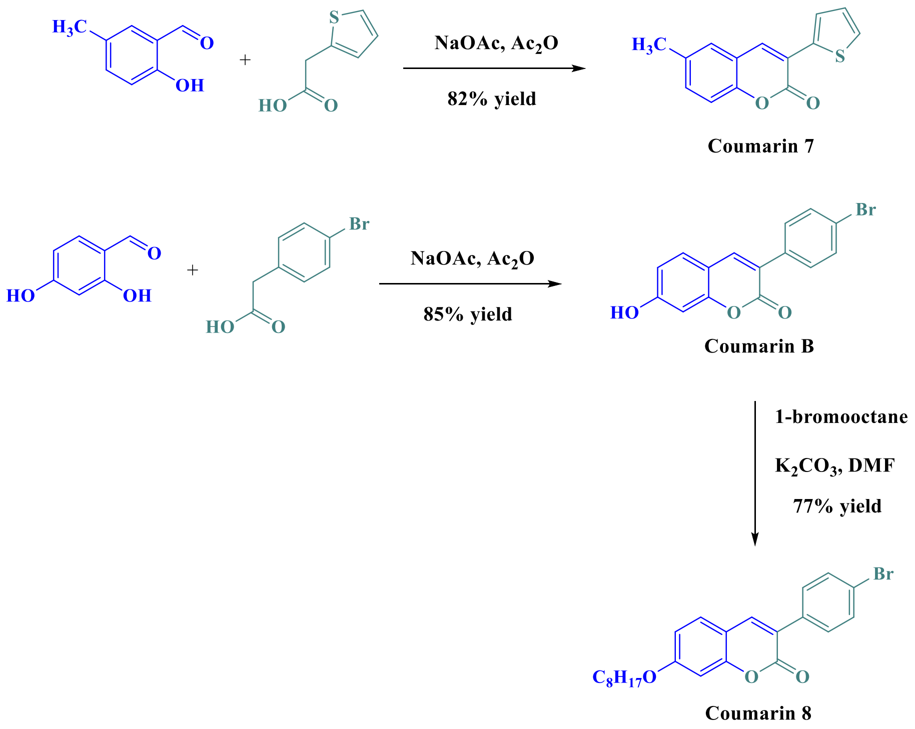

Figure S4. Synthetic route to **Coumarins 7 and 8**.

*Synthesis of 6-methyl-3-(thiophen-2-yl)-2H-chromen-2-one (**Coumarin 7**)*

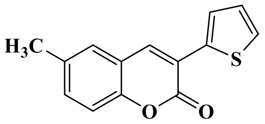

Chemical Formula: $C_{14}H_{10}O_2S$
Exact Mass: 242.0402
Molecular Weight: 242.2920

Anhydrous CH_3COONa (2.65 g, 32.07 mmol, M = 82.03 g/mol), 2-thiopheneacetic acid (3.28 g, 23.05 mmol, M = 142.17 g/mol), and 5-methyl-2-hydroxybenzaldehyde (3.14 g, 23.05 mmol, M = 136.15 g/mol) was refluxed in Ac_2O (20 mL) for 2 h. The solution was cooled to room temperature during which time a precipitate formed. It was filtered off, washed several times with water, pentane and dried under vacuum (4.58 g, 82 % yield). ^1H NMR ($CDCl_3$) δ : 2.40 (s, 3H), 7.20 (t, 1H, J = 3.9 Hz), 7.35 (d, 1H, J = 8.4 Hz), 7.44 (d, 1H, J = 10.0 Hz), 7.58 (s, 1H), 7.69 (d, 1H, J = 4.1 Hz), 7.86 (d, 1H, J = 3.74 Hz), 8.51 (s, 1H) ; ^{13}C NMR ($CDCl_3$) δ : 22.4, 115.7, 119.0, 120.4, 126.6, 127.3, 128.0, 128.7, 132.4, 134.0, 135.3, 136.1, 150.3, 159.1 ; HRMS (ESI MS) m/z: theor: 243.0474 found: 243.0476 ($[M+H]^+$ detected).

Synthesis of 3-(4-bromophenyl)-7-hydroxy-2H-chromen-2-one

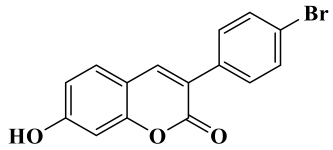

Chemical Formula: $C_{15}H_9BrO_3$
Exact Mass: 315.9735
Molecular Weight: 317.1380

Anhydrous CH_3COONa (2.65 g, 32.07 mmol, M = 82.03 g/mol), 4-bromophenylacetic acid (4.96 g, 23.05 mmol, M = 215.05 g/mol), and 2,4-dihydroxybenzaldehyde (3.18 g, 23.05 mmol, M = 138.12 g/mol) was refluxed in Ac_2O (20 mL) for 2 h. The solution was cooled to room temperature during which time a precipitate formed. It was filtered off, washed several times with water, pentane and dried under vacuum (6.21 g, 85 % yield). ^1H NMR (DMSO-d_6) δ : 1.62 (brs, 1H, OH), 7.09 (dd, 1H, J = 8.4 Hz, J = 2.1 Hz), 7.16 (d, 1H, J = 2.1 Hz), 7.55 (d, 1H, J = 8.4 Hz), 7.57-7.58 (m, 4H), 7.80 (s, 1H); ^{13}C NMR (DMSO-d_6) δ : 21.1, 110.1, 117.3, 118.6, 123.3, 126.6, 128.6, 130.1, 131.7, 133.4, 139.2, 153.1, 154.1, 159.9, 168.6; HRMS (ESI MS) m/z: theor: 316.9808 found: 316.9811 ($[M+H]^+$ detected).

*Synthesis of 3-(4-bromophenyl)-7-(octyloxy)-2H-chromen-2-one (**Coumarin 8**)*

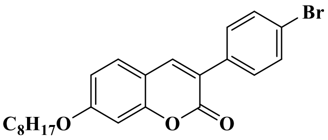

Chemical Formula: $C_{23}H_{25}BrO_3$
Exact Mass: 428.0987
Molecular Weight: 429.3540

3-(4-Bromophenyl)-7-hydroxy-2*H*-chromen-2-one (3 g, 9.45 mmol, M = 317.14 g/mol), potassium carbonate (5 g, 36.18 mmol, M = 138.20 g/mol) and bromooctane (2.74 g, 14.2 mmol, M = 193.12 g/mol) wee dissolved in DMF (50 mL) and the solution was stirred at 80°C overnight. The solvent and the excess of bromooctane were removed under reduced pressure. The residue was purified by column chromatography (SiO_2) using a gradient of solvent from pentane to DCM (3.12 g, 77 % yield). ^1H NMR ($CDCl_3$) δ : 0.82 (t, 3H, J = 6.2 Hz), 1.23-1.34 (m, 8H), 1.39 (qt, 2H, J = 7.8 Hz), 1.75 (qt, 2H, J = 7.0 Hz), 3.96 (t, 2H, J = 6.5 Hz), 6.77-6.81 (m, 2H), 7.35 (d, 1H, J = 8.5 Hz), 7.47-7.53 (m, 4H), 7.69 (s, 1H); ^{13}C NMR ($CDCl_3$) δ : 14.1, 22.6, 26.0, 29.0, 29.2, 29.3, 31.8, 68.8, 100.9, 113.0, 113.4, 122.6, 123.4, 128.9, 130.0, 131.6, 134.0, 140.1, 155.4, 166.7, 162.5; HRMS (ESI MS) m/z: theor: 429.1060 found: 429.1056 ($[M+H]^+$ detected).
